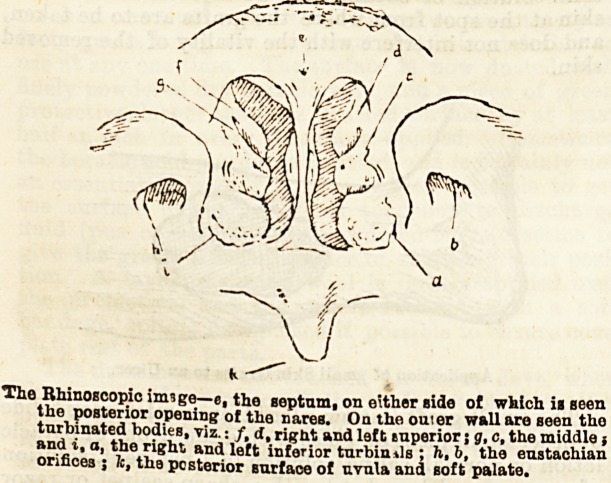# Rhinoscopy

**Published:** 1893-06-10

**Authors:** P. Watson Williams

**Affiliations:** Physician to the Throat Department, Bristol Royal Infirmary


### THE HOSPITAL CLINIC.

*[The Editor will be glad to receive offers of co-operation and contributions from members of the profession. All letters should be addressed to* THE EDITOR, THE LODGE, PORCHESTER SQUARE, LONDON, W.]

### RHINOSCOPY—IV.

EXAMINATION OF THE NOSE—(*continued*).

P. WATSON WILLIAMS, M.D.Lond., Physician to the Throat Department, Bristol Royal Infirmary.

Posterior rhinoscopy, or the examination of the naso-pharynx and posterior nares from behind is much more difficult of accomplishment. The patient sits with the head held vertically, and the mouth wide open.

In a few cases we find that the tongue is sufficiently under control of the patient, lying on the floor of the mouth, to render a tongue depressor unnecessary, but as a rule we have to depress the tongue. In doing so we must remember to place the depressor just beyond the arch of the tongue and no further. If the depressor is not far enough back, the tongue bulges back and occludes the view, whereas if the depressor is too far back retching results.

The patient should be directed to breath quietly. Every care should be taken to avoid "flurrying" him, and difficulties are often introduced by giving directions as to the manner of breathing. If the soft palate does not soon relax, and the patient continues to breath through the mouth only, it is well to direct him to *breathe in* through the nose, and nasal respiration will probably be unconsciously continued. If the space between the soft palate and the posterior pharyngeal wall is too narrow to permit a view of the naso-pharynx being obtained, it will be necessary to use a soft palate retractor. Voltolini's is the simplest for examination purposes, while Cresswell Baber's modification of White's self-retaining palate retractor is most serviceable for operating.

But before using a retractor the fauces and posterior surface of the palate should be anæsthetised with cocaine, and not infrequently the use of cocaine will obviate the necessity for retractors, especially where the fauces are very irritable, and there is much gagging in spite of every precaution against putting the tongue depressor too far back. Above all, avoid touching the fauces and posterior pharyngeal wall in introducing the rhinoscopic mirror.

The Rhinoscopic image—*e*, the septum, on either side of which is seen the posterior opening of the nares. On the outer wall are seen the turbinated bodies, viz.: *f*, *d*, right and left superior; *g*, *c*, the middle; and *i*, *a*, the right and left inferior turbinals; *h*, *b*, the eustachian orifices; *k*, the posterior surface of uvula and soft palate.

Having successfully passed the mirror behind the soft palate, it should be tilted forward until the central posterior margin of the septum is seen. On carefully but rapidly tilting the mirror first to one side and then the other the posterior nares are seen, and projecting inward toward the septum, the posterior extremities of the inferior and middle turbinated bodies. The inferior turbinated bodies appear as small greyish masses resting on the lower margin of the choanæ, and the middle turbinated as pinkish grey. Sometimes we can observe the superior and middle meatuses, never the inferior meatus. Near the upper margin the superior turbinated bodies can just be dimly seen. Below them we see the posterior surface of the soft palate and uvula. By turning the mirror still further out, we obtain a view of the lower ends of the Eustachian tubes with their orifices, and behind them the depressions of Rosenmüller's fossæ. Again, finding the septum the mirror is tilted back so as to successively bring into view the vault and the posterior wall of the naso-pharynx, with the collection of adenoid tissue called Luschka's tonsils. By mentally collecting the various parts thus brought into view we obtain a knowledge of the condition of the region as a whole. It is hardly necessary to add that the time occupied in examining the region must necessarily be short, as the soft palate is sure to retract after a short interval. Consequently it is better to make several examinations, instead of attempting a lengthy and complete examination without withdrawing and reintroducing the mirror.

In examining very young children for post-nasal growths it is often impossible to get a view of the naso-pharynx, and the parts should be digitally explored by introducing the forefinger behind the soft palate and feeling the posterior margin of the septum, the vault, and posterior wall.

In making such a digital exploration the finger should be guarded by a thick rubber finger guard, or by a gag or spatula held between the teeth by the free hand.

## Figures and Tables

**Figure f1:**